# Altered extracellular matrix correlates with an immunosuppressive tumor microenvironment and disease progression in younger adults with oral cavity squamous cell carcinoma

**DOI:** 10.3389/fonc.2024.1412212

**Published:** 2024-06-18

**Authors:** Leonard E. Estephan, Gaurav Kumar, Matthew Stewart, Raphael Banoub, Alban Linnenbach, Larry A. Harshyne, Ubaldo E. Martinez-Outschoorn, My G. Mahoney, Joseph M. Curry, Jennifer Johnson, Andrew P. South, Adam J. Luginbuhl

**Affiliations:** ^1^ Department of Otolaryngology - Head and Neck Surgery, Thomas Jefferson University Hospitals, Philadelphia, PA, United States; ^2^ Sidney Kimmel Cancer Center, Thomas Jefferson University Hospitals, Philadelphia, PA, United States; ^3^ Department of Cancer Biology, Thomas Jefferson University, Philadelphia, PA, United States; ^4^ Department of Internal Medicine, Cleveland Clinic Foundation, Cleveland, OH, United States; ^5^ Department of Pharmacology, Physiology and Cancer Biology, Thomas Jefferson University, Philadelphia, PA, United States; ^6^ Department of Medical Oncology, Thomas Jefferson University Hospitals, Philadelphia, PA, United States

**Keywords:** oral cavity squamous cell carcinoma (OSCC), age-related tumor aggressiveness, tumor microenvironment, immunosuppression, extracellular matrix

## Abstract

**Introduction:**

Oral cavity squamous cell carcinoma (OSCC) occurs most frequently in patients >60 years old with a history of tobacco and alcohol use. Epidemiological studies describe increased incidence of OSCC in younger adults (<45 years). Despite its poor prognosis, knowledge of OSCC tumor microenvironment (TME) characteristics in younger adults is scarce and could help inform possible resistance to emerging treatment options.

**Methods:**

Patients with OSCC were evaluated using TCGA-HNSC (n=121) and a stage and subsite-matched institutional cohort (n=8) to identify differential gene expression focusing on the extracellular matrix (ECM) and epithelial-mesenchymal transition (EMT) processes in younger (≤45 years) vs. older adults (≥60 years). NanoString nCounter analysis was performed using isolated total RNA from formalin-fixed paraffin-embedded (FFPE) tumor samples. Stained tumor slides from young and old OSCC patients were evaluated for CD8^+^ T-cell counts using immunohistochemistry.

**Results:**

Younger OSCC patients demonstrated significantly increased expression of ECM remodeling and EMT process genes, as well as TME immunosuppression. Gene set enrichment analyses demonstrated increased ECM pathways and concurrent decreased immune pathways in young relative to old patients. Transcripts per million of genetic markers involved in ECM remodeling including LAMB3, VCAN, S100A9, COL5A1, and ITGB2 were significantly increased in tumors of younger vs. older patients (adjusted p-value < 0.10). Young patient TMEs demonstrated a 2.5-fold reduction in CD8^+^ T-cells as compared to older patients (p < 0.05).

**Conclusion:**

Differential gene expression impacting ECM remodeling and TME immunosuppression may contribute to disease progression in younger adult OSCC and has implications on response to evolving treatment modalities, such as immune checkpoint inhibitor therapy.

## 1 Introduction

Arising mostly in the mucosal linings of the oral cavity, pharynx, and larynx, head and neck squamous cell carcinoma (HNSCC) represents an aggressive multi-factorial disease that accounts for over 650,000 new cases annually, with a mortality rate of approximately 50–60% per year. Specifically, oral cavity squamous cell carcinoma (OSCC) is primarily a disease of older adults, occurring mostly in patients older than age 60 with a demonstrated history of tobacco and/or alcohol use (1–4). Younger cancer patients, by virtue of their age, have typically had a shorter durations of toxin and chemical exposure than their older counterparts raising the question of other etiologies of disease. Although human papillomavirus (HPV) has a well-described role in squamous cell carcinoma of the oropharynx in younger and middle-aged adults, it is not currently considered a driver of OSCC and data from HPV-positive patients is not included in this manuscript (2, 5).

Whether younger OSCC patients display a more aggressive phenotype with a poorer prognosis and a lower 3–5-year survival rate as compared to older OSCC patients remains controversial. A meta-analysis of 23,382 collective patients with mostly T1/T2 disease demonstrated no difference in disease-specific survival for patients aged 18–40 compared with those >40 years of age, yet multiple single-center studies report worsened survival in younger patients (4, 6–8). Oral cavity cancer is aggressive regardless of age, and includes a similar 5-year overall survival rate to several other head and neck cancers with HPV-negative status (9). This disease may confer an added complexity and severity when presenting in younger patients with no antecedent risk factors. In younger patients that lack extensive exposure to alcohol and tobacco, unique genetic predispositions may contribute to more severe OSCC disease. Notably, previous studies have found local recurrences and distant metastases are more prevalent in younger as compared to older patients (2). Based on its unclear etiology and potentially more aggressive clinical course, OSCC in younger patients may even be considered a distinct clinical entity compared to OSCC in older adults.

Treatment for OSCC has focused on multimodal therapy comprised of surgery, radiation, and chemotherapy. The poor overall survival of this cancer has prompted the search for new treatment strategies and paradigms. Recent success has been found with immune checkpoint inhibitors (ICI), specifically monoclonal antibodies targeting PD-1, which have been approved in the setting of recurrent or metastatic disease. There is intense interest in incorporating these agents into the care of locally advanced disease both to improve patient survival and potentially decrease toxicity. Patients who achieve immune engagement with ICIs can experience prolonged responses, though this occurs in less than 20% of patients. The toxicity profile is favorable and thus far does not appear to be additive with other modalities used in the locally advanced setting (10, 11). In order to optimize the use of novel immunotherapeutic agents we must better understand the enormous diversity of HNSCC. Tumors present with varying degrees of fibrosis, immune infiltrate, and alterations in the tumor microenvironment (TME) that may impact primary response to ICI and facilitate secondary resistance to these agents (12, 13).

Collectively, younger adults with or without risk factors who develop OSCC pose difficult questions of disease course and sequelae of treatment. Oral cavity tumors in this age group have been relatively understudied. Earlier reports have mainly focused on the epidemiological association of younger OSCC patients with prognosis and survival, but lack the mechanistic investigations necessary to understand the etiology of OSCC in this specific population (1–4, 14, 15). To address this, the present study utilizes multiple differential gene expression analysis methods to investigate genetic characteristics of younger and older OSCC cohorts that we hypothesize may contribute tumor aggressiveness and progression in the younger OSCC subset. Aggressiveness is defined in this manuscript as carrying elements of tumor infiltration and known histopathologic features that contribute to worsened disease-specific survival as it pertains to OSCC.

## 2 Materials and methods

### 2.1 TCGA data source

The RNA sequencing data as raw count matrix, and clinical data for 506 HNSCC patients from The Cancer Genome Atlas (TCGA) was obtained using the ‘TCGAbiolinks’ package in R/Bioconductor (16). Clinical data was stratified (HPV-negative and oral cavity subsite) resulting in 121 patients (n=17 [ ≤ 45 years old], n=104 [≥60 years old]) used for subsequent analysis. Upper and lower age cutoffs were selected to isolate distinct OSCC cohorts for gene expression analysis. The ages of <45 years for the young cohort and >60 years for the older cohort were selected as cutoff criteria based on epidemiological reports of OSCC disease incidence and criteria used in prior studies pertaining to this topic (1–4).

### 2.2 NanoString nCounter gene expression assay

This study was approved by the Thomas Jefferson University Institutional Review Board. Eight subjects from our institutional biobank were identified (4 younger and 4 older adults), and RNA from their formalin-fixed paraffin-embedded (FFPE) tumor samples was isolated using an miRNeasy FFPE kit (Qiagen, Hilden, Germany). Age at the time of surgery was used to define our young (≤45 years) and old (≥60 years) cohorts (**Table 1**). Briefly, after excess paraffin was trimmed from each sample, the residual was removed by using deparaffinization solution and incubation at 56^°^C for 3 minutes. At room temperature, buffer PKD was added; samples were transferred to bead mill tubes containing 2.8 mm ceramic beads. Tissue was disrupted using a Bead Ruptor at 5 m/sec for 15 seconds, followed by icing for 45 seconds (Omni International, GA, USA) three separate times. Lysates were centrifuged at 11,000 xg 1 min. Proteinase K was added to the clear phase (~24 mAnson Units/ml), followed by serial incubations at 56^°^C and 80^°^C with intermittent vortexing. After centrifugation at 11,000 xg 1 minute, the lower phase was recovered, further clarified by centrifugation, then treated with DNaseI. Buffer RBC and ethanol were added for optimal binding conditions, and samples were applied to RNeasy MinElute spin columns. After rinsing with buffer RPE, total RNA was eluted in 20–30 µl of RNase-free water. FFPE RNA sample QC was determined using an Agilent TapeStation (Agilent Technoligies, CA, USA). DV_200_ determinations for percentage of total RNA fragments >200 nt, averaged 42 (range 18–63). An nCounter analysis system was utilized to run the PanCancer IO 360 Panel for 16 immuno-oncology pathways and bioprocesses (NanoString, WA, USA). NanoString technology is considered advantageous over next-generation sequencing and polymerase chain reaction as it is rapid, technically simple, and does not require nucleic acid amplification. Additionally, this tool has recently been implicated in diagnostic methods in cancers of the breast and lung, leukemia, and lymphoma (17).

**Table 1:** Demographic and clinical characteristics of OSCC patient cohorts.

	Old Cohort (n=4)	Young Cohort (n=4)
Age at Surgery (range in years)	71–84	32–44
Sex (Male: Female)	2:2	3:1
Self-Reported Race		
Caucasian	2	4
Indian	1	0
Asian	1	0
Oral Cavity Tumor Subsite		
Mobile Tongue	3	1
Ventral Tongue/FOM	1	1
Lower Gingiva	0	2
HPV Status		
Negative	4	4
Positive	0	0
T Stage		
T2	1	1
T3/T4a	3	3
N Stage		
N2b	1	3
N2c	1	1
N3	2	0
M Stage		
M0	4	4
Evidence of PNI		
Present	4	2
Absent	0	2
Evidence of LVI		
Present	3	2
Absent/Indeterminate	1	2
Recurrence		
Yes	2	2
No	2	2
Reported Alcohol Use		
≥14 drinks/week	0	0
<14 drinks/week	4	4
Reported Smoking History		
≥10 pack years	3	2
<10 pack years	1	2
Current Status		
Alive	1	1
Deceased	3	3

OSCC, oral cavity squamous cell carcinoma; FOM, floor of mouth; HPV, human papillomavirus; PNI, perineural invasion; LVI, lymphovascular invasion.

### 2.3 Differential expression analysis

Raw counts were utilized from the TCGA-HNSC cohort and the NanoString nCounter PanCancer IO 360 Panel was applied to our institutional biobank samples. Consisting of 770 genes, the PanCancer IO 360 Panel combines vital components involved in the complex interplay between the tumor microenvironment and the immune response in cancer. Before differential expression, batch effects or sample heterogeneity was tested using iSeqQC (18). Differential gene expression was performed between young and older adults using the DESeq2 package in R/Bioconductor (19). Genes were considered differentially expressed if they had an adjusted p-value ≤ 0.10. All plots were constructed using R/Bioconductor.

### 2.4 Functional annotations

The DESeq2 test statistic was used as a ranking metric to perform Gene Set Enrichment Analysis (GSEA) in pre-ranked mode, with genes having zero base mean or “NA” test statistic values filtered out to avoid providing numerous duplicate values. The GSEA was performed against hallmark gene sets from MSigDB collections (20). Additionally, an analysis using the Database for Annotation, Visualization, and Integrated Discovery (DAVID) was performed on our list of differentially expressed genes between younger vs. older patients to identify biological processes modulated between these cohorts (21). Further, IPA software was used (Ingenuity system, Qiagen, CA, USA) to evaluate functional changes in the young adults and network analysis was performed using Cytoscape (22).

### 2.5 Immunohistochemistry analysis

Ten additional patients (n=5 each of younger and older patients) with HPV-negative OSCC and available FFPE samples were utilized. Patients were matched based on site and stage of their tumor. Tissue preserved in FFPE was obtained from surgically resected specimens and underwent staining with monoclonal antibodies directed against CD8 (anti-CD8 (SP57) rabbit monoclonal primary antibody; Ventana Medical Systems, AZ, USA). The slides were digitally scanned at 20x magnification using the iScan HT whole-slide image scanner (Roche, Switzerland). Fully automated detection was performed on a Ventana Discovery Ultra System (Ventana Medical Systems, AZ, USA).

## 3 Results

### 3.1 Etiology of OSCC tumorigenesis in young patients

**Figure 1:**
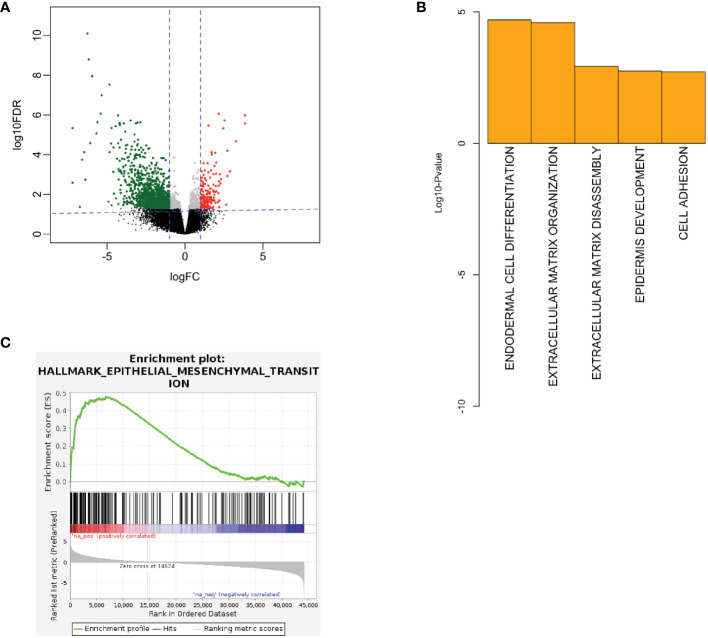
**(A)** Volcano plot showing RNA expression between young and old patients in TCGA. Here, red color denotes up-regulated genes in young and green denotes down-regulated genes in young patients. **(B)** Biological processes being up-regulated in the young patients in TCGA. **(C)** GSEA showing EMT processes to be up-regulated in the young TCGA patients (FDR q-value < 0.01, NES = 2.49). EMT, epithelial-mesenchymal transition, FC, fold-change, FDR, false discovery rate, GSEA, gene set enrichment analysis, IHC, immunohistochemistry, NES, normalized enrichment score, TCGA, the cancer genome atlas.

To interrogate sequencing data for a signal of cancer aggressiveness in young vs. older OSCC patients, we first utilized the TCGA-HNSC cohort. Differential RNA expression analysis was performed between OSCC from younger patients (n=17) and older patients (n=104) using DESeq2 (19). Statistical power was determined to be adequate for subsequent analysis with the size of each respective cohort. Here, we observed 1856 genes to be differentially expressed, where 214 genes were up-regulated, and 1642 were down-regulated in young patients (**Figure 1A**). Next, using the differentially expressed genes, we performed gene ontology analysis to evaluate the biological processes implicated in the younger patients using DAVID (21). Here, we observed up-regulation of ECM structural organization (**Figure 1B**) in the young oral cavity patients when compared to the older patients. Furthermore, GSEA analysis using ranked gene lists showed epithelial-mesenchymal transition (EMT) processes to be among the most up-regulated hallmarks in the younger cohort (**Figure 1C**). These results implicate the ECM and EMT processes in promoting disease progression in the OSCC tumors of young adults. Patient identifiers from TCGA and a list of differentially expressed genes can be found in the **Supplementary File**.

### 3.2 Role of ECM modifications in promoting aggressiveness in young adult tumors

**Figure 2:**
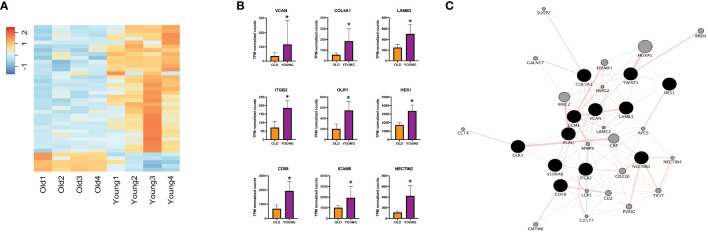
**(A)** Heatmap showing expression of differentially expressed genes between young and old patients using nCounter. **(B)** NanoString nCounter gene expression profiles of different genes of interest. **(C)** Network analysis including genes of interest showing physical interaction with ECM-related genes. *Adjusted p-value < 0.10 for young compared to old. ECM, extracellular matrix, TPM, transcripts per million.

Next, to validate our findings from the TCGA-HNSC cohort, we utilized an institutional OSCC cohort. Here, FFPE tumor blocks were obtained for 8 site and stage-matched patients (n=4 each for young and old patients) and RNA was extracted to perform NanoString nCounter gene expression assays using the PanCancer IO 360 Panel. A summary of patient age (at time of surgery), self-reported race, sex, tumor subsite within the oral cavity, HPV status, TNM staging, evidence of perineural invasion, evidence of lymphovascular invasion, disease recurrence, reported alcohol use, reported smoking history, and current status of patients from the institutional cohort can be found in **Table 1**. Patient RNA expression for the 770 genes of the PanCancer IO 360 Panel was obtained and differential expression was performed between young and old OSCC patients using DESeq2. Here, we found 40 genes to be differentially expressed in the young patients compared to older patients (34 up-regulated, 6 down-regulated) (**Figure 2A**). Next, functional annotations were performed on the differentially expressed genes, confirming our TCGA results. We found genes involved in cell adhesion and ECM processes to be up-regulated in young vs. older patients. These data correspond to our TCGA-HNSC observations, and strongly suggest that ECM remodeling is a key biological process contributing to aggressiveness in the OSCC tumors of young patients. NanoString outputs are available in the public repository FigShare or are available upon request.

To characterize the key mediators that might be promoting ECM remodeling processes in young OSCC patients, we performed functional annotation analysis using IPA software. We identified key genes including VCAN, S100A9, COL5A1, LAMB3, ITGB2, OLR1, HES1, CD58, TWIST1, and NECTIN2 to be of high-interest due to their described role in functions of disease progression in multiple cancers (**Figure 2B**) (23–32). To evaluate their interaction in ECM remodeling, we constructed a network map using Cytoscape (**Figure 2C**), which showed physical interaction of LAMB3 with key ECM-regulating genes such as COL5A1, PLAU, ITGB2, and ECM1 (33–36).

### 3.3 Immunosuppressed tumor microenvironment in younger patients

**Figure 3:**
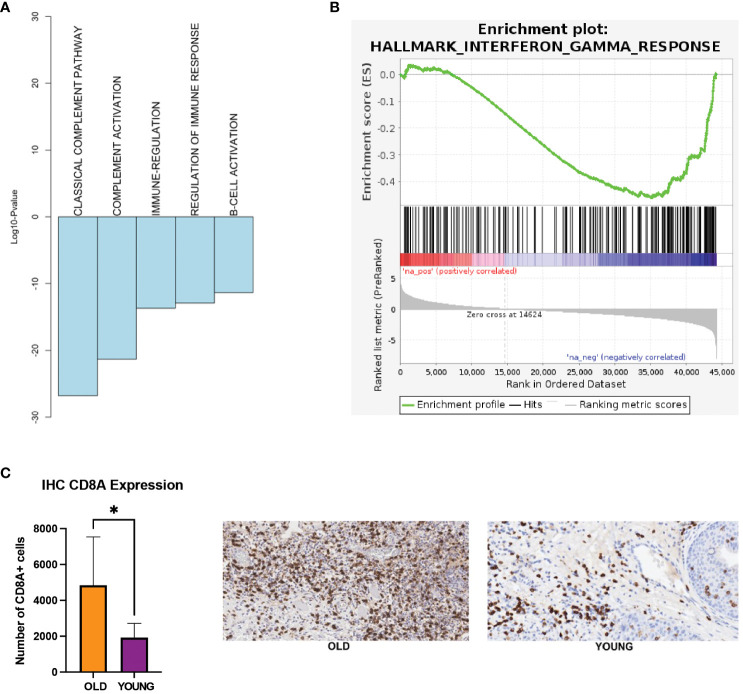
**(A)** Biological processes being down-regulated in the young patients in TCGA. **(B)** GSEA showing interferon gamma response processes to be down-regulated in the young patients in TCGA (FDR q-value = 0.01, NES = -1.61). **(C)** IHC expression of CD8 T-cells. *p-value < 0.05 for young compared to old patients. EMT, epithelial-mesenchymal transition; FDR, false discovery rate; GSEA, gene set enrichment analysis; IHC, immunohistochemistry; NES, normalized enrichment score; TCGA, the cancer genome atlas.

Our analysis of the TCGA-HNSC and nCounter gene expression panel showed down-regulation of immune-related processes in the young OSCC patients. Here, functional annotation analysis of the TCGA-HNSC cohort demonstrated down-regulation of complement and B-cell activations, as well as the interferon gamma response processes in the young OSCC patients (**Figures 3A, B**). To assess the immune cell presence in younger patients, we performed immunohistochemistry (IHC) of CD8^+^ T-cells on FFPE samples of younger and older OSCC patients (n=10; 5 each for younger and older patients). Here, we observed a significant reduction (2.5-fold change; p<0.05) of CD8^+^ T-cell infiltration in the young OSCC tumors (**Figure 3C**). Collectively, these data suggest that younger OSCC patients have a more immunosuppressive TME, where the stromal cells may dynamically modulate the microenvironment to favor tumor aggressiveness in this age group.

### 3.4 ECM promotion of immunosuppression in the TME

**Figure 4:**
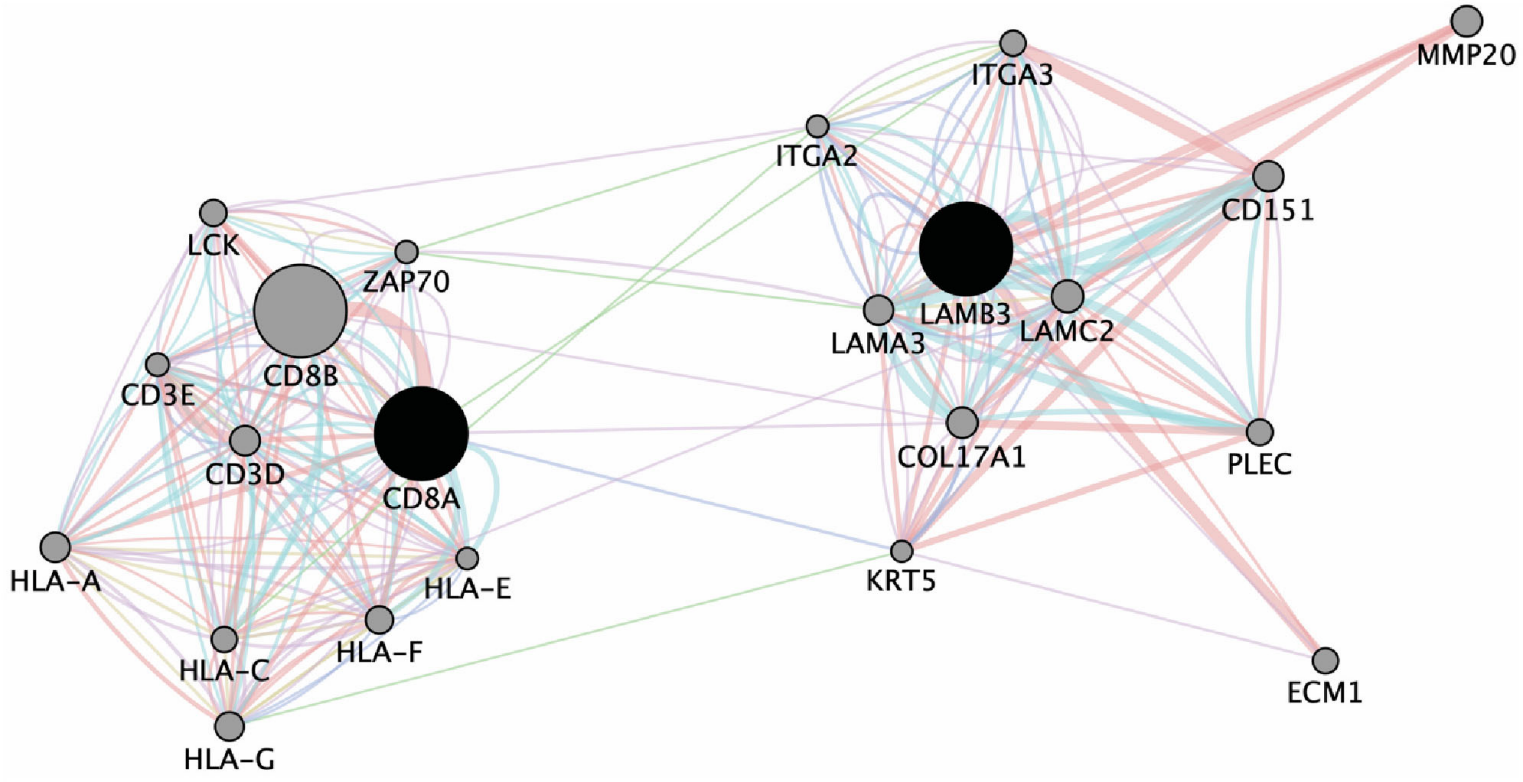
Network map demonstrating interaction of LAMB3 and CD8A.

To discern whether LAMB3 might be regulating the immunosuppression in the TME of young OSCC adults, we evaluated the correlation of these genes with immune-regulation using an in-silico approach. We did so by performing network analysis of LAMB3 to identify its interactions with the CD8A gene. As demonstrated in **Figure 4**, LAMB3 is predicted to interact with CD8A, CD3D, and CD3E. Other immune and ECM-specific genes such as ECM1, ITGA2, COl17A1, KRT5, and HLA are also shown to be amongst the node genes.

## 4 Discussion

The present study identifies ECM remodeling and EMT processes as central in promoting increased aggressiveness in younger adult OSCC tumors. The ECM is an essential component of the TME, as increased ECM component deposition and crosslinking contributes to tumor progression and is a major barrier to effective checkpoint immunotherapy treatment (37, 38). Previous reports discuss the pathological role of increased ECM remodeling in HNSCC relative to normal tissue (39–41). However, to our knowledge, this is the first study to describe ECM remodeling processes as having an enhanced differential expression in younger vs. older OSCC patients. Specifically, we report increased LAMB3 activity, suggesting its role as a primary gene impacting the ECM in younger adults. LAMB3 is known to exhibit tumorigenic effects in multiple types of cancer, including HNSCC (42). However, its role in promoting tumorigenesis particularly in young OSCC patients has not been previously well-described.

As it pertains to tumor resistance in immune checkpoint inhibition (ICI) therapy, increased ECM deposition in young OSCC patients may drastically impact prognosis. Previous work has demonstrated the deleterious effects of aberrant ECM remodeling on ICI therapy in several types of cancer. In a study of metastatic melanoma samples, Hugo et al. assessed the transcriptomes and tumor mutanomes of pretreatment melanoma biopsies to better define the role of genetics in resistance to anti-PD-1 therapy. They reported that tumors with innate resistance to immunotherapy displayed transcriptional signatures of upregulated genes implicated in ECM remodeling in addition to cellular adhesion, mesenchymal transition, and angiogenesis (38). Separately, Peng et al. identified the negative consequences of extensive tumor collagen deposition on anti-PD-1/PD-L1 effectiveness in lung tumors. Increased ECM resulted in elevated LAIR1 activity which mechanistically exhausts T-cells by way of tyrosine phosphatase SHP-1, rendering ICI therapy less effective. As a proof-of-concept, when this group reduced collagen deposition in their model through LOXL2 suppression, both T-cell exhaustion and anti-PD-1 resistance were diminished (37). Given these findings, the increased ECM activity described in our young OSCC cohort may contribute to ICI treatment resistance of such patients.

Extracellular matrix remodeling not only contributes to tumor progression and resistance to therapy, but also impacts TME immune cell presence and activity. It is known that the TME is comprised of several cell populations, including cancer and stromal cells, in addition to non-cell ECM components. A complex interplay exists between cellular stromal subpopulations and tumor cells communicated through various secreted cytokines, chemokines, growth factors and ECM proteins (39). Our findings suggest that in young OSCC patients there is a degree of immune suppression relative to older individuals, which we believe is due in part to increased ECM remodeling processes. In assessing our nCounter gene expression panel, we observed up-regulation of genes associated with fibroblasts (LAMB3 and VCAN) in the tumors of our younger patients. In addition, CD8^+^ T-cells were not as prevalent in younger patients demonstrated through IHC. Mechanistically, several specific effects of ECM processing on immune cell function have been proposed. The ECM dynamically modulates the physical organization, signaling cascades, and cellular constituents in the tumors for both exclusion and inactivation of T-cells (37, 43–47). Exclusion of T-cells might be regulated through haptotaxis signals from altered ECM, where instead of entering the TME, T-cells migrate along ECM-rich encapsulation of tumors due to the gradients of substrate rigidity and adhesion molecules such as aligned collagen fiber. Stiffened ECM can induce poor diffusion that can enhances glycolytic metabolism and acidification, which suppresses the activation of T-lymphocytes through the specific interaction between V-domain immunoglobulin suppressor of T-cell activation and co-inhibitory receptor P-selectin glycoprotein ligand-1 in acidic TMEs. Proteins of the ECM are known to contribute to the direct regulation of T-lymphocytes. As reported, CD8^+^ T-cells are suppressed by collagen through LAIR1 and tyrosine phosphatase SHP-1, and high molecular weight hyaluronic acid could enhance the activity of regulatory T-cells *in vitro* (37, 43–47). Concurrent increases in matrix remodeling paralleled by suppressed TME immune activity may promote both disease progression and tumor aggressiveness.

Our results suggest that LAMB3 may be up-regulated in the TME of the younger OSCC patients and act as part of a gene network along with ITGA2 and LAMC2. In a recently published study of pancreatic ductal adenocarcinoma (PDAC), Islam et al. proposed that these three genes potentiate disease progression and severity. Levels of gene mRNA expression were significantly higher in PDAC tissue as compared to normal tissue, and expression levels were associated with increased pathological tumor stage. Disease-specific and overall survival were significantly reduced in patients with high LAMB3, ITGA2, and LAMC2 expression as compared to patients with low expression (31). Similar to PDAC, these ECM-related genes may contribute to disease aggressiveness in the young OSCC population.

Besides LAMB3, prior reports discuss other significantly up-regulated genes from our study as they pertain to various cancers. Mitsui et al. studied VCAN and its potential implications in clear cell renal cell carcinoma (ccRCC). This group concluded that VCAN was associated with poor prognosis through induction of tumor development by reducing TNF-mediated cellular apoptosis (28). Besides ccRCC, VCAN has been described for its potential negative role in malignancy of the prostate, colon, and ovary (48–50). Similarly, COL5A1 was found to negatively contribute to ovarian cancer, as described in a recent study by Zhang et al. This group demonstrated elevated expression of COL5A1 in ovarian cancer cells, while knockdown of this gene reduced cellular capabilities to proliferate and migrate. Additionally, this protein was found to be overexpressed specifically in cells resistant to paclitaxel, which was significantly improved following gene silencing (51). The remainder of genes described in our study have also been implicated in several types of cancer, but an extensive discussion of each is beyond the scope of this manuscript.

Converse to an apparent upregulation of such genes, we observed a diminished CD8^+^ T-cell presence in the TME of young patients. Natural killer (NK) cells have been shown to secrete FLT3L, which is a potent driver of dendritic cell (DC) activation and antigen presentation ability. Further, DC-CD8 interactions within the TME are vital for proper immune surveillance. The implications of theoretically diminished FLT3L pathway in young patients with OSCC may be drastic. Bickett et al. used the TCGA to describe an increased 5-year overall survival in HNSCC patients with increased FLT3L and FLT3 (ligand receptor) expression, demonstrating the consequences of a depleted NK cell response (52). Taken together, the immune suppression in the TME of our young patients, as evidenced by decreased CD8^+^ cell presence, may contribute to enhanced tumor aggressiveness through obtunded immune cell signaling, activity, and surveillance. Not only are the implications for aggressive tumor behavior elucidated in these findings, but also the major implications of immunotherapy resistance. As ICI treatment becomes a front-line approach either in the neoadjuvant or definitive setting, we need to be thoughtful about defining patients’ TME to inform us about known resistance patterns. In addition, further work aimed at identifying strategies to improve therapeutic efficacy is vitally important for patients with OSCC.

Our study is not without certain limitations. Tumor tissue utilized for the NanoString nCounter analysis was obtained from a cohort of 4 younger and 4 older patients from our institution. Ideally, this matched cohort could have been enlarged to include additional patients and possibly strengthen our findings. Additionally, the tumor samples available for our IHC quantification of CD8^+^ T-cells within the TME were of institutional patients of the same age restrictions but were separate from the NanoString group. Under ideal conditions, these samples would have come from the same matched cohort, however this was not possible due to tissue availability. Lastly, this study of young OSCC patients provides evidence of differential expression for genetic markers known to be involved in cancer progression, however future studies are required to further elucidate mechanisms of relatively poor prognosis in this population.

## 5 Conclusions

This study suggests that younger adults with OSCC have a TME with depletion of effector immune cells and enhanced ECM remodeling processes, contributing to increased disease aggressiveness. As a downstream effect, this patient population may also suffer from increased resistance to certain therapeutic options, such as immunotherapy. Further work is required to expand upon these initial findings and define specific mechanistic pathways leading to worsened prognosis for OSCC patients of young age.

## Data availability statement

The datasets presented in this study can be found in online repositories. The names of the repository/repositories and accession number(s) can be found below: https://figshare.com/, https://figshare.com/s/152292bb44d76d45c2ad.

## Ethics statement

The studies involving humans were approved by Thomas Jefferson University Hospital Institutional Review Board. The studies were conducted in accordance with the local legislation and institutional requirements. The participants provided their written informed consent to participate in this study.

## Author contributions

LE: Investigation, Writing – original draft, Writing – review & editing, Data curation. GK: Writing – review & editing, Data curation, Formal analysis, Writing – original draft. MS: Data curation, Investigation, Writing – review & editing. RB: Data curation, Investigation, Writing – review & editing. ALi: Data curation, Writing – review & editing. LH: Writing – review & editing. UM-O: Writing – review & editing. MM: Writing – review & editing. JC: Writing – review & editing. JJ: Visualization, Writing – review & editing. AS: Visualization, Writing – review & editing. ALu: Writing – review & editing, Methodology, Supervision, Visualization.
