# Supplementary material for: Altered extracellular matrix correlates with an immunosuppressive tumor microenvironment and disease progression in younger adults with oral cavity squamous cell carcinoma
Source: Front Oncol. 2024 Jun 18;14:1412212. doi: 10.3389/fonc.2024.1412212 (PMC11217481; doi:10.3389/fonc.2024.1412212)
Supplement: Supplementary file 1 [file DataSheet_1.docx]

Supplementary Table 1. Young vs. Old Differentially Expressed Genes

| Gene Symbol |
| --- |
| \| TNMD \| \| --- \| \| GCLC \| \| ENPP4 \| \| CFTR \| \| ICA1 \| \| SLC7A2 \| \| CD38 \| \| PDK4 \| \| ACSM3 \| \| TSPOAP1 \| \| SOX8 \| \| KRT33A \| \| ABCC8 \| \| CX3CL1 \| \| ETV1 \| \| CCL26 \| \| USH1C \| \| SCIN \| \| PROM1 \| \| NOS2 \| \| DNAH9 \| \| SLC13A2 \| \| TKTL1 \| \| FMO3 \| \| CAMK1G \| \| TFAP2B \| \| DLEC1 \| \| ADAM22 \| \| PAX7 \| \| SYT7 \| \| SEMA3B \| \| LTF \| \| CLDN11 \| \| CLCA4 \| \| IGF1 \| \| VSIG2 \| \| PLEKHB1 \| \| NRXN3 \| \| GCLM \| \| IKZF2 \| \| MYOC \| \| SLC18A1 \| \| C6 \| \| SOX30 \| \| CDH10 \| \| USH2A \| \| TG \| \| ADAM28 \| \| JADE2 \| \| ADRB1 \| \| TNFRSF17 \| \| LMO3 \| \| NEXMIF \| \| LAMA3 \| \| LY75 \| \| CBLN4 \| \| LAMC2 \| \| PRDM6 \| \| ARSF \| \| SLC6A16 \| \| CCN5 \| \| DMRT3 \| \| EYA2 \| \| SNCAIP \| \| BCAS1 \| \| COL17A1 \| \| TLE2 \| \| SLC9A3 \| \| MPPED2 \| \| REEP1 \| \| PYGM \| \| MAOB \| \| FGF10 \| \| FSTL3 \| \| ST6GALNAC1 \| \| MYO3B \| \| LNX1 \| \| SIDT1 \| \| PANX2 \| \| FNDC8 \| \| GSDMB \| \| ST6GAL1 \| \| SNCB \| \| WSCD2 \| \| SEMA3C \| \| ATP12A \| \| RARB \| \| SPAG6 \| \| FKBP6 \| \| BRINP1 \| \| BPIFB2 \| \| CLUL1 \| \| CDH17 \| \| DCT \| \| EPHA6 \| \| SLC4A4 \| \| LRP2 \| \| C1QTNF3 \| \| OPRK1 \| \| EPYC \| \| SLC27A5 \| \| SLCO1A2 \| \| EFR3B \| \| MECOM \| \| PHACTR3 \| \| TESC \| \| ANKRD24 \| \| LYZ \| \| FETUB \| \| NRCAM \| \| SLC26A4 \| \| IL5RA \| \| TF \| \| SLC7A8 \| \| PHGDH \| \| GABRP \| \| FMO2 \| \| CYP26A1 \| \| CRTAC1 \| \| MYO3A \| \| CRISP3 \| \| MISP \| \| CECR2 \| \| DERL3 \| \| TPTEP1 \| \| RSPH14 \| \| TIMP3 \| \| HMOX1 \| \| APOL4 \| \| MLC1 \| \| GZMH \| \| GZMB \| \| COCH \| \| PLEK2 \| \| TCL1A \| \| MMP9 \| \| RIMS4 \| \| BMP7 \| \| NTSR1 \| \| SPEF1 \| \| PLCB4 \| \| PAK5 \| \| CST4 \| \| WFDC2 \| \| GPR143 \| \| BMX \| \| PHEX \| \| ITIH6 \| \| FGF14 \| \| CCL22 \| \| FA2H \| \| NECAB2 \| \| CRYM \| \| TOX3 \| \| TMC5 \| \| AP3B2 \| \| OCA2 \| \| PDGFRL \| \| ZDHHC2 \| \| EYA1 \| \| PLAT \| \| IL7 \| \| AMH \| \| FCER2 \| \| KIR3DX1 \| \| TJP3 \| \| SIGLEC8 \| \| CD79A \| \| CEACAM5 \| \| FAM83E \| \| SLC5A5 \| \| PDE4C \| \| COMP \| \| HPN \| \| PTN \| \| LFNG \| \| CRHR2 \| \| CYP3A5 \| \| SERPINE1 \| \| MOGAT3 \| \| SFRP4 \| \| AGR2 \| \| SLC1A1 \| \| OGN \| \| PTGR1 \| \| TYRP1 \| \| PIP5K1B \| \| ATRNL1 \| \| TLX1 \| \| FGF8 \| \| WNT3 \| \| TRIM16L \| \| ALDH3A1 \| \| CCL2 \| \| KRT32 \| \| RND2 \| \| HLF \| \| GABRA4 \| \| ODAM \| \| ANXA10 \| \| TRIM2 \| \| FAM149A \| \| DDX25 \| \| B3GAT1 \| \| CCKBR \| \| FOLR3 \| \| ELMOD1 \| \| POU2AF1 \| \| ACSS3 \| \| PRR4 \| \| PRMT8 \| \| MANSC1 \| \| CHPT1 \| \| GYS2 \| \| GCNT2 \| \| FAM184A \| \| ULBP1 \| \| PACRG \| \| SMOC2 \| \| CLIC5 \| \| ENPP5 \| \| C7 \| \| NME5 \| \| PCDHB3 \| \| THBS4 \| \| SLC27A6 \| \| SLC12A7 \| \| HRG \| \| GRK7 \| \| SERPINI2 \| \| LRRC31 \| \| PFKFB4 \| \| COL7A1 \| \| ROPN1B \| \| ABCC5 \| \| ARHGEF26 \| \| PLCH1 \| \| DNAH1 \| \| EVA1A \| \| DNAH6 \| \| OTX1 \| \| SLC9A2 \| \| SLC5A7 \| \| DLX2 \| \| LCT \| \| KYNU \| \| KIAA1324 \| \| FBXO2 \| \| PRG4 \| \| CFHR3 \| \| HPCAL4 \| \| ACTL8 \| \| PLA2G2D \| \| CR2 \| \| FMO6P \| \| FASLG \| \| TNFSF4 \| \| MUC5B \| \| COLEC11 \| \| CASC1 \| \| LRMP \| \| MYB \| \| SLC16A7 \| \| RARRES1 \| \| DNAH7 \| \| ECRG4 \| \| GALNT12 \| \| BCL11A \| \| CFAP58 \| \| SFRP5 \| \| GRIA2 \| \| TEX11 \| \| CLU \| \| TBX4 \| \| PRB2 \| \| BCL2L14 \| \| CSMD2 \| \| RASL11A \| \| LAX1 \| \| LY9 \| \| DNAI1 \| \| DBH \| \| G0S2 \| \| CTCFL \| \| PTGIS \| \| CDH26 \| \| C20orf85 \| \| PCK1 \| \| ATP8A1 \| \| CRISP2 \| \| BTN1A1 \| \| SPDEF \| \| TEKT3 \| \| C1orf61 \| \| SRMS \| \| SLC25A23 \| \| GRIA3 \| \| TGM3 \| \| BMP2 \| \| PCSK2 \| \| LRRN4 \| \| BPIFB1 \| \| AMOT \| \| MCF2L \| \| STATH \| \| WNK4 \| \| HSPA2 \| \| PLAAT1 \| \| TSPAN8 \| \| VIL1 \| \| FGL2 \| \| VPREB3 \| \| GNAZ \| \| MGAT3 \| \| APOBEC3F \| \| TBC1D27P \| \| LRRC4 \| \| FEZF1 \| \| PDE11A \| \| ACKR4 \| \| AJUBA \| \| FOXA1 \| \| CDO1 \| \| QRICH2 \| \| FOXJ1 \| \| FGF13 \| \| PRRG3 \| \| GDPD2 \| \| KIF1A \| \| GDF15 \| \| IQCN \| \| OLFM1 \| \| CYP2E1 \| \| PNPLA7 \| \| SMPDL3B \| \| BPIFA2 \| \| C1QL1 \| \| IDO1 \| \| PPP1R1B \| \| SLC6A11 \| \| DDC \| \| FCRL2 \| \| AL139352.1 \| \| KANK4 \| \| SLC14A2 \| \| PDE6A \| \| TPTE2 \| \| STOML3 \| \| IRS4 \| \| PDE6B \| \| PLAAT4 \| \| MYH11 \| \| ZDHHC8P1 \| \| NTS \| \| DYDC2 \| \| MRO \| \| TRPM1 \| \| REG4 \| \| GSTM3 \| \| HMGCS2 \| \| VTCN1 \| \| SAA2 \| \| ERN2 \| \| SOX5 \| \| KLRD1 \| \| PRH2 \| \| CLDN10 \| \| MSI1 \| \| HNF1A \| \| RNFT2 \| \| FAM71F1 \| \| NT5E \| \| MRAP2 \| \| EPHA7 \| \| ELF5 \| \| GLS2 \| \| CAPN9 \| \| GPR55 \| \| PAX3 \| \| HTR2B \| \| SERPINE2 \| \| WNT10A \| \| DGKB \| \| CHAD \| \| SCN2A \| \| ERMN \| \| NR5A1 \| \| CTSV \| \| IL33 \| \| DMRT1 \| \| CD72 \| \| IGFBPL1 \| \| GCM1 \| \| C6orf52 \| \| FGFBP2 \| \| TMPRSS4 \| \| CYP1B1 \| \| SIX3 \| \| CH25H \| \| RBP4 \| \| ZNF365 \| \| FAM117B \| \| CILP \| \| FGF5 \| \| MTTP \| \| SHISAL1 \| \| COL2A1 \| \| PPFIA2 \| \| TPH2 \| \| PTPRQ \| \| AMDHD1 \| \| N4BP2L1 \| \| MORN3 \| \| SLAIN1 \| \| SRRM4 \| \| ZIC5 \| \| TTC6 \| \| NOVA1 \| \| SYT16 \| \| WARS \| \| SLC25A47 \| \| DUOX2 \| \| SLC27A2 \| \| FGF7 \| \| CYP11A1 \| \| CYP1A1 \| \| CCDC33 \| \| NTRK3 \| \| CDH13 \| \| SKAP1 \| \| RHBDL3 \| \| SECTM1 \| \| CBLN2 \| \| FBN3 \| \| PRDM16 \| \| PADI1 \| \| PGD \| \| DMRTA2 \| \| CYP4B1 \| \| GPA33 \| \| MAEL \| \| ADCY10 \| \| NR1I3 \| \| FCRL5 \| \| ANXA9 \| \| SELENBP1 \| \| SUSD4 \| \| EPHX1 \| \| REN \| \| SYT2 \| \| ABHD1 \| \| ANKRD53 \| \| ST6GAL2 \| \| THSD7B \| \| GULP1 \| \| PLA1A \| \| ALDH1L1 \| \| UCN2 \| \| STXBP5L \| \| ILDR1 \| \| TM4SF19 \| \| MUC4 \| \| PLAC8 \| \| GLRA3 \| \| GZMA \| \| BHMT \| \| TIMD4 \| \| GABRB2 \| \| CPLX2 \| \| PSD2 \| \| GFRA3 \| \| LGSN \| \| SCUBE3 \| \| TCTE1 \| \| PRSS35 \| \| GABRR1 \| \| CLVS2 \| \| SLC2A12 \| \| PNLDC1 \| \| NLGN4X \| \| RAB19 \| \| SLC16A2 \| \| ZNF711 \| \| GPC3 \| \| PMP2 \| \| DPYS \| \| RSPO2 \| \| NTRK2 \| \| PLPPR1 \| \| CRB2 \| \| ASTN2 \| \| CACNA1B \| \| CDHR1 \| \| PLEKHS1 \| \| GAS2 \| \| LRRC4C \| \| SAA4 \| \| SCGB1A1 \| \| GRIK4 \| \| DUSP15 \| \| GPHA2 \| \| TLCD3B \| \| CNKSR2 \| \| CNTN5 \| \| GPM6A \| \| CNDP1 \| \| PRSS23 \| \| FREM2 \| \| EPS8 \| \| NR3C2 \| \| AKR1C2 \| \| TMEM163 \| \| TMEM178A \| \| GRIA4 \| \| IGSF10 \| \| BMP3 \| \| JAKMIP1 \| \| ACOXL \| \| SYCP2L \| \| ADGRF1 \| \| FAM81B \| \| C16orf89 \| \| FAM92B \| \| KCNJ16 \| \| CHST9 \| \| GPR15 \| \| CYP4Z2P \| \| GAL3ST2 \| \| ENPP3 \| \| TDH \| \| GBP5 \| \| SORBS2 \| \| CHODL \| \| TMPRSS15 \| \| NCAM2 \| \| L3MBTL4 \| \| WNT7A \| \| APCDD1 \| \| CCDC144B \| \| RAB6B \| \| CA10 \| \| FZD7 \| \| SAXO1 \| \| AFF2 \| \| NAT2 \| \| GNA14 \| \| WIF1 \| \| ALX3 \| \| CLDN8 \| \| FGF18 \| \| RAB11FIP1 \| \| GLYATL2 \| \| LHFPL4 \| \| SUSD3 \| \| C1orf158 \| \| TSPAN18 \| \| SLC34A2 \| \| DRC1 \| \| RHPN1 \| \| PPP1R9A \| \| DYNC1I1 \| \| NPM2 \| \| FGF17 \| \| CLIC6 \| \| TUBBP5 \| \| CLDN14 \| \| PADI4 \| \| IRX6 \| \| CES5A \| \| CELF3 \| \| ADGRG5 \| \| SPON2 \| \| LRRC36 \| \| PIP \| \| NPR2 \| \| TNFRSF13C \| \| FNDC5 \| \| CILP2 \| \| TFF3 \| \| TFF1 \| \| TMPRSS3 \| \| RSPH1 \| \| SLC37A1 \| \| LRRC71 \| \| AZGP1 \| \| SCGB3A1 \| \| IKZF3 \| \| ITGA5 \| \| AQP5 \| \| ALOX15 \| \| SCIMP \| \| CCDC42 \| \| ZG16B \| \| BSND \| \| PRKAA2 \| \| RBP7 \| \| TTLL10 \| \| CCDC27 \| \| AKNAD1 \| \| GBP4 \| \| KCNT2 \| \| VCAM1 \| \| TRIM58 \| \| AXDND1 \| \| SNED1 \| \| KLHDC8A \| \| PKDCC \| \| OXER1 \| \| PIGR \| \| FCAMR \| \| CAPN13 \| \| CCDC74A \| \| SLC16A14 \| \| CFAP221 \| \| CCDC140 \| \| SPRR3 \| \| C1orf189 \| \| NPPC \| \| LMOD1 \| \| ELF3 \| \| RETNLB \| \| DPPA2 \| \| SERPINI1 \| \| ICA1L \| \| CD200R1 \| \| FAM86KP \| \| SLC6A20 \| \| GRIK3 \| \| DNALI1 \| \| KLF15 \| \| SCRG1 \| \| HPGD \| \| RANBP3L \| \| SCGB3A2 \| \| HTR4 \| \| CDC20B \| \| ENPP6 \| \| PI16 \| \| SMAD5-AS1 \| \| HEY1 \| \| SHH \| \| C8orf48 \| \| HNF4G \| \| SBSPON \| \| DIRAS2 \| \| NIPSNAP3B \| \| ALDH1A1 \| \| AL353743.1 \| \| TRPV6 \| \| RNF183 \| \| PCDH19 \| \| CLDN3 \| \| WNK2 \| \| AQP7 \| \| ARMC3 \| \| DEUP1 \| \| TSHR \| \| OTX2 \| \| STOX1 \| \| NDRG2 \| \| BTNL9 \| \| PDZRN4 \| \| IL25 \| \| PPP1R14D \| \| GABRB3 \| \| RAG1 \| \| ZMAT1 \| \| MC4R \| \| CHRFAM7A \| \| PLEKHA7 \| \| ACSM1 \| \| PLIN1 \| \| MESP1 \| \| MYO1A \| \| SCG5 \| \| TGM6 \| \| MS4A8 \| \| CCDC178 \| \| EVA1C \| \| UGT1A6 \| \| PRR15L \| \| KATNAL2 \| \| LPO \| \| GPD1 \| \| TMC4 \| \| TMIGD2 \| \| PLIN4 \| \| GPT \| \| ANGPTL4 \| \| TEKT1 \| \| KRT24 \| \| SOST \| \| BATF2 \| \| PNOC \| \| FOXI1 \| \| FAM107A \| \| MOBP \| \| CMAHP \| \| PHYHIP \| \| CCDC110 \| \| CAVIN2 \| \| CHRM1 \| \| GDNF \| \| UGT3A2 \| \| KCTD19 \| \| LRP1B \| \| CA7 \| \| GSTM4 \| \| CXXC4 \| \| IL12A \| \| FSTL5 \| \| SFTPB \| \| NTSR2 \| \| AR \| \| GSG1L \| \| IL13 \| \| P2RY12 \| \| C22orf15 \| \| GP2 \| \| SCN9A \| \| CCDC8 \| \| NLGN1 \| \| MYO7B \| \| RNF150 \| \| CST1 \| \| LRRN2 \| \| KRT78 \| \| KRT86 \| \| MZB1 \| \| KRT4 \| \| LONRF2 \| \| HSD17B13 \| \| TMC7 \| \| SIX2 \| \| DLGAP1 \| \| SLC26A5 \| \| KCNS3 \| \| BFSP2 \| \| CEL \| \| LILRP2 \| \| PDGFD \| \| PLAC1 \| \| NRTN \| \| KCNMB3 \| \| KCNG3 \| \| SMR3B \| \| CLDN20 \| \| UGT2B7 \| \| LRG1 \| \| KRT15 \| \| KRT13 \| \| NEUROD2 \| \| NMUR1 \| \| NMRAL2P \| \| WDR87 \| \| PCDHB1 \| \| AQP4 \| \| MIR31HG \| \| CYP4F11 \| \| CD8B \| \| MACROD2 \| \| IL17D \| \| CTSW \| \| THEMIS \| \| NBEA \| \| ADH6 \| \| FRG2C \| \| RXFP4 \| \| ADCY5 \| \| ABCD2 \| \| TUBB8P12 \| \| SLC2A14 \| \| SAA1 \| \| AGR3 \| \| CSPG4 \| \| UGT2A1 \| \| MUC13 \| \| TDRD12 \| \| KCNH6 \| \| GPR160 \| \| SLCO4C1 \| \| PIFO \| \| SPERT \| \| C9orf131 \| \| TLR1 \| \| AC020659.1 \| \| SNX31 \| \| ZNF80 \| \| SLC16A11 \| \| CHRNA9 \| \| C11orf45 \| \| MFSD4A \| \| KLK15 \| \| LEP \| \| WDR49 \| \| BTC \| \| PDZK1 \| \| DNAH12 \| \| CATSPERD \| \| AMZ1 \| \| CADM2 \| \| VWA3A \| \| CHRNA7 \| \| KCNE3 \| \| LINC02694 \| \| ASCL3 \| \| C11orf16 \| \| JAKMIP2 \| \| ACBD7 \| \| PRR18 \| \| GNG7 \| \| C15orf56 \| \| LINC00303 \| \| MUC20 \| \| SCN4B \| \| PRDM16-DT \| \| UMODL1 \| \| CD19 \| \| ERICH5 \| \| OR2T8 \| \| ZBED2 \| \| RPRM \| \| SAMD12 \| \| NAALADL2 \| \| KCNJ10 \| \| GLDC \| \| CA8 \| \| ERBB4 \| \| DYNAP \| \| TMEM52 \| \| AC114812.1 \| \| ERICH3 \| \| ZFP42 \| \| AC020907.1 \| \| PXT1 \| \| NSUN7 \| \| SLITRK4 \| \| CIITA \| \| ALOX15B \| \| ARL14 \| \| NLRP11 \| \| C1orf194 \| \| NRXN1 \| \| ZNF648 \| \| TH \| \| SLC9A4 \| \| ALX1 \| \| ITPRID1 \| \| BHLHA15 \| \| OR52N4 \| \| ADIPOQ \| \| AC004832.1 \| \| SLC25A41 \| \| CFAP65 \| \| SAGE1 \| \| SOX2 \| \| MAB21L2 \| \| FDCSP \| \| SLC2A4 \| \| CLDN7 \| \| FAM181B \| \| ENPP7 \| \| RGMA \| \| NLRP10 \| \| KIAA2012 \| \| CACNB4 \| \| LHFPL1 \| \| SPNS3 \| \| VCX \| \| HS3ST4 \| \| TSPAN10 \| \| KCNB2 \| \| GALR2 \| \| RGS6 \| \| PLCXD3 \| \| TCHHL1 \| \| PCP4 \| \| GAS6 \| \| CSMD1 \| \| TMEM119 \| \| CHST6 \| \| CCDC60 \| \| GBP6 \| \| TENT5C \| \| KLRC4 \| \| PSG9 \| \| MACC1 \| \| B3GALT5 \| \| TUBA8 \| \| MAATS1 \| \| PNMA3 \| \| FAM3B \| \| SRARP \| \| KCNH8 \| \| TREML3P \| \| RPL7AP28 \| \| C1QTNF12 \| \| CCSER1 \| \| NCMAP \| \| PTGER4P2 \| \| C6orf58 \| \| SLITRK6 \| \| FAM167A-AS1 \| \| LRRC26 \| \| B3GALT5-AS1 \| \| TRARG1 \| \| TCEAL2 \| \| CLCNKB \| \| ANKS1B \| \| SGCZ \| \| FLRT2 \| \| MIXL1 \| \| LINC00482 \| \| PRKN \| \| HS6ST3 \| \| METTL7A \| \| SV2B \| \| DLK1 \| \| OLFML2A \| \| PBX1 \| \| SYN3 \| \| CCDC190 \| \| TMPRSS11B \| \| CALHM1 \| \| UBL4B \| \| CYP4Z1 \| \|  \| \| CYP4X1 \| \| AKAP14 \| \| RGS7BP \| \| ANKRD20A5P \| \| CYP4F3 \| \| HPDL \| \| MPPED1 \| \| FOXI2 \| \| FOXE3 \| \| EFCAB6 \| \| CCK \| \| SLIT1 \| \| AKR1C1 \| \| C12orf74 \| \| ATP13A5 \| \| CYP26C1 \| \| TCL6 \| \| C2orf88 \| \| DNAJB13 \| \| DIPK1C \| \| TTC24 \| \| FYB2 \| \| DMBT1 \| \| OR7D2 \| \| TPRG1 \| \| RTP5 \| \| NWD1 \| \| HEPACAM2 \| \| PLA2G2A \| \| IL17REL \| \| OR7A5 \| \| C15orf62 \| \| SBK1 \| \| GPAT2P2 \| \| JAKMIP3 \| \| CLEC2A \| \| IGHV1OR15-9 \| \| SERPINA5 \| \| CFAP77 \| \| NKAIN2 \| \| BCL2L15 \| \| SNTN \| \| ZDHHC11 \| \| BEND4 \| \| C9orf152 \| \| MSANTD1 \| \| KIR2DL4 \| \| ANKDD1B \| \| CGB5 \| \| APOD \| \| FAM25G \| \| KRT77 \| \| NUGGC \| \| ALKAL2 \| \| FAM180A \| \| CXCL17 \| \| PTPRT \| \| AKR1C3 \| \| COLCA1 \| \| KIF19 \| \| CTSE \| \| LINC00615 \| \| GRM7 \| \| ADH7 \| \| ASMT \| \| ESRRG \| \| ADH1B \| \| LINC00173 \| \| ALKAL1 \| \| HLA-DQA1 \| \| LAMB3 \| \| GPRACR \| \| FAM163B \| \| DTHD1 \| \| ACSL5 \| \| C6orf141 \| \| RAMP2-AS1 \| \| CYP2B6 \| \| GGT3P \| \| CYP2F1 \| \| AC034105.1 \| \| C5orf56 \| \| KCNMB2 \| \| AC068051.1 \| \| MFAP5 \| \| CR1L \| \| STPG3 \| \| CYP2A13 \| \| PRB3 \| \| KEL \| \| DLGAP2 \| \| AKR1B10 \| \| CYP2A7 \| \| BPIFA1 \| \| TMEM116 \| \| AL354714.1 \| \| B3GNT6 \| \| ITGBL1 \| \| C20orf203 \| \| RD3 \| \| SH2D1B \| \| AKR1C4 \| \| FAM3D \| \| LPA \| \| COLGALT2 \| \| MUC2 \| \| RYR3 \| \| CES1 \| \| CCDC152 \| \| L1CAM \| \| NOS1AP \| \| RNU5B-2P \| \| AL626787.1 \| \| TAS2R2P \| \| LINC00970 \| \| LINC00862 \| \| CCDC162P \| \| RBM20 \| \| ADD3-AS1 \| \| CCDC160 \| \| TCEAL5 \| \| NEU4 \| \| NAP1L6 \| \| CXorf65 \| \| TCEA3 \| \| LINC02731 \| \| HLA-DOA \| \| HLA-DMA \| \| CYP21A1P \| \| NXPE2 \| \| SLC44A4 \| \| PSORS1C1 \| \| C6orf15 \| \| ACOXL-AS1 \| \| IGKV1OR2-3 \| \| MALRD1 \| \| IGFL2 \| \| IGFL4 \| \| KRT40 \| \| CD177 \| \| PSG5 \| \| C2orf91 \| \| TTLL10-AS1 \| \| MT1A \| \| INSYN1 \| \| EXOC3L4 \| \| KRTAP10-2 \| \| MUC19 \| \| ARRDC5 \| \| C16orf96 \| \| TTC23L \| \| AC105345.1 \| \| IGLL3P \| \| TMEM211 \| \| ZDHHC11B \| \| CD200R1L \| \| PRSS50 \| \| RNU6-481P \| \| DIO1 \| \| IGKV5-2 \| \| IGKV6-21 \| \| IGKV3D-20 \| \| IGKV3D-11 \| \| IGLV8-61 \| \| IGLV4-60 \| \| IGLV10-54 \| \| IGLV1-51 \| \| IGLV1-50 \| \| IGLV1-47 \| \| IGLV7-46 \| \| IGLV5-45 \| \| IGLV1-44 \| \| IGLV2-33 \| \| IGLV3-32 \| \| IGLV3-27 \| \| IGLV3-19 \| \| IGLV2-14 \| \| IGLV3-12 \| \| IGLV3-10 \| \| IGLV4-3 \| \| IGLC2 \| \| IGHA2 \| \| IGHG4 \| \| IGHA1 \| \| IGHG1 \| \| IGHD \| \| IGHJ2 \| \| IGHJ1 \| \| IGHV6-1 \| \| IGHV1-2 \| \| IGHV2-5 \| \| IGHV3-7 \| \| IGHV3-11 \| \| IGHV3-16 \| \| IGHV1-18 \| \| IGHV3-20 \| \| IGHV3-23 \| \| IGHV1-24 \| \| IGHV2-26 \| \| IGHV3-33 \| \| IGHV4-34 \| \| IGHV3-38 \| \| IGHV4-39 \| \| IGHV3-48 \| \| IGHV3-49 \| \| IGHV5-51 \| \| IGHV1-58 \| \| IGHV3-66 \| \| IGHV5-78 \| \| SNORD67 \| \| KLHL23 \| \| ASIC3 \| \| AC008481.2 \| \| GSTM2 \| \| KRT222 \| \| CFL1P2 \| \| AKR1B10P1 \| \| AC004129.1 \| \| KLRK1 \| \| UBD \| \| UCA1 \| \| TMEM213 \| \| LINC02026 \| \| AGGF1P1 \| \| COLCA2 \| \| FOXI3 \| \| PLIN5 \| \| LRRC37A11P \| \| HMGN2P15 \| \| CAPN14 \| \| AP002358.1 \| \| LINC00612 \| \| SMTNL1 \| \| GRID2IP \| \| LINC00269 \| \| MUC5AC \| \| AKR1C7P \| \| MIR99AHG \| \| LINC00189 \| \| KRT18P3 \| \| ANKRD20A11P \| \| GCGR \| \| TTC34 \| \| AL109918.1 \| \| AL021407.1 \| \| LINC01644 \| \| AC110619.1 \| \| Z97206.1 \| \| SNORA11 \| \| C1orf229 \| \| MYBPHL \| \| SNORA79B \| \| IGLV9-49 \| \| AC009988.1 \| \| AC005165.1 \| \| IGHV3-64 \| \| AC106786.1 \| \| MIR503HG \| \| TSSC2 \| \| LINC01983 \| \| AC073365.1 \| \| AC142381.1 \| \| AL390728.3 \| \| LINC02561 \| \| WARS2-IT1 \| \| AL391427.1 \| \| AC131097.2 \| \| CHL1-AS2 \| \| IGHV4-59 \| \| C4B \| \| AC103563.1 \| \| IGKV1D-27 \| \| IGHV3-74 \| \| Z84484.1 \| \| LEMD1-DT \| \| PCOLCE-AS1 \| \| AL355482.1 \| \| AC114812.2 \| \| COL4A2-AS2 \| \| AP001347.1 \| \| FTH1P22 \| \| LINC00092 \| \| CT62 \| \| IGKV1OR9-2 \| \| SFTA1P \| \| AL354707.1 \| \| GBP1P1 \| \| AC005237.1 \| \| AC064875.1 \| \| AL008633.1 \| \| IGHV3-72 \| \| LINC02036 \| \| DBH-AS1 \| \| MTND4P23 \| \| LINC00626 \| \| RPS27P16 \| \| ABHD11-AS1 \| \| AC005515.1 \| \| UQCRFS1P1 \| \| TEX22 \| \| LINC02519 \| \| ARHGAP26-AS1 \| \| AL122058.1 \| \| LINC01876 \| \| OR8T1P \| \| ADAM1B \| \| LINC00323 \| \| AC087499.2 \| \| AC012512.1 \| \| MEIS1-AS3 \| \| THORLNC \| \| ATE1-AS1 \| \| SLC8A1-AS1 \| \| AL157935.1 \| \| SLAMF6P1 \| \| AC067942.1 \| \| HPN-AS1 \| \| AC012501.2 \| \| LINC01268 \| \| AL162414.1 \| \| AL391056.1 \| \| LINC01068 \| \| AL358394.1 \| \| DNMBP-AS1 \| \| MYL12BP2 \| \| DNAJB3 \| \| TARID \| \| AC097713.1 \| \| AL355607.1 \| \| TXNP5 \| \| COL6A4P2 \| \| CYP4F29P \| \| IGKV3D-7 \| \| GAS2L1P2 \| \| UGT1A2P \| \| LINC01546 \| \| AP006222.1 \| \| MIR34AHG \| \| AC093159.1 \| \| AC005042.2 \| \| AC245100.3 \| \| CES1P1 \| \| LINC02575 \| \| THRB-AS1 \| \| AL033523.1 \| \| AC104653.1 \| \| LINC01344 \| \| AC007038.1 \| \| AL356056.1 \| \| LINC00582 \| \| KRT16P4 \| \| MYOSLID \| \| AC013460.1 \| \| CXCR2P1 \| \| STEAP3-AS1 \| \| LINC01315 \| \| AL590666.2 \| \| AL357143.1 \| \| AC093585.1 \| \| ANKRD36BP2 \| \| PPIAP39 \| \| KRT16P5 \| \| AL022316.1 \| \| LINC02525 \| \| LINC00595 \| \| U73166.1 \| \| ANKRD30BP3 \| \| AC104461.1 \| \| AC024084.1 \| \| PRB4 \| \| AL035252.2 \| \| STMND1 \| \| SNX18P13 \| \| AC096637.2 \| \| LINC01508 \| \| AL031666.1 \| \| AL445426.1 \| \| AC106799.1 \| \| IGKV1OR2-108 \| \| AC103563.3 \| \| AC005392.2 \| \| FAM27C \| \| LINC01249 \| \| AL139288.1 \| \| SLC47A1P2 \| \| LUARIS \| \| FSIP2-AS1 \| \| LINC01704 \| \| AL033397.1 \| \| AL354993.1 \| \| ABCA9-AS1 \| \| CHODL-AS1 \| \| AL035701.1 \| \| Z93403.1 \| \| AL359551.1 \| \| PRH1 \| \| PSG1 \| \| AC078845.1 \| \| LINC01697 \| \| LINC01873 \| \| IGHV3-43 \| \| AL158166.2 \| \| AL589935.1 \| \| LINC02576 \| \| AC004990.1 \| \| FRG2FP \| \| LINC00342 \| \| RPL23AP87 \| \| AL354707.2 \| \| AL663074.1 \| \| RNF224 \| \| CHRM3-AS2 \| \| AL139002.1 \| \| AC008063.2 \| \| RPL17P11 \| \| AL035414.1 \| \| LINC02609 \| \| SLC26A4-AS1 \| \| LINC01122 \| \| NRAD1 \| \| IGHV3OR16-10 \| \| LINC01237 \| \| AC103563.7 \| \| AL390778.2 \| \| AC009970.1 \| \| IGKV3OR2-268 \| \| LINC02535 \| \| AL356867.1 \| \| LINC01731 \| \| AL451069.3 \| \| AC009264.1 \| \| LINC01914 \| \| AC007278.1 \| \| LINC01250 \| \| LNCTAM34A \| \| AC002076.1 \| \| AC005064.1 \| \| FOXP4-AS1 \| \| SLC25A25-AS1 \| \| LINC01524 \| \| SHISA8 \| \| AC099654.2 \| \| AC006159.1 \| \| AL355990.2 \| \| LINC01871 \| \| LINC00494 \| \| AC007389.3 \| \| AP001625.2 \| \| LINC01828 \| \| TM4SF19-AS1 \| \| LINC01564 \| \| AL354766.2 \| \| AL109946.1 \| \| ELFN1-AS1 \| \| SCN1A-AS1 \| \| AC110995.1 \| \| AC019197.1 \| \| AC019117.2 \| \| FAM157A \| \| LINC02195 \| \| TCF3P1 \| \| ZNF853 \| \| AC079780.1 \| \| ARHGEF38 \| \| LINC01829 \| \| ANKRD20A7P \| \| AC024560.1 \| \| AL359979.2 \| \| MIR600HG \| \| CLCA4-AS1 \| \| RPL12P12 \| \| GLIS3-AS1 \| \| IGHJ3P \| \| BX276092.7 \| \| AL034397.2 \| \| DCLRE1CP1 \| \| LINC01684 \| \| SHISA9 \| \| AF064858.2 \| \| GSTA7P \| \| AL139039.3 \| \| AL365259.1 \| \| AC022034.1 \| \| AL035425.1 \| \| LINC01238 \| \| AL606970.4 \| \| OR2I1P \| \| DDX39BP2 \| \| CD81-AS1 \| \| OR2W3 \| \| IFNWP19 \| \| LINC02006 \| \| U8 \| \| U8 \| \| LLPH-DT \| \| CASTOR3 \| \| AC125618.1 \| \| IGKV1D-8 \| \| IGKV1-6 \| \| IGKV1-37 \| \| PRKAG2-AS1 \| \| IGKV3-20 \| \| IGKV1D-33 \| \| AC069431.1 \| \| AL512306.2 \| \| IGKV1-17 \| \| AC034238.1 \| \| C1QTNF9 \| \| IGKV1-8 \| \| IGKV1-16 \| \| LINC02042 \| \| RPL22P2 \| \| UGT1A9 \| \| ADAMTS9-AS1 \| \| IGKV1D-16 \| \| SYNPR-AS1 \| \| CA15P1 \| \| RARRES2P1 \| \| IGKV1-9 \| \| AC104411.1 \| \| AL627309.5 \| \| IGKV1-33 \| \| RPS20P33 \| \| AC093789.1 \| \| AC121764.1 \| \| UGT1A8 \| \| IGKV1-39 \| \| LINC01206 \| \| UGT1A10 \| \| AC012501.3 \| \| IGKV1D-43 \| \| CD200R1L-AS1 \| \| SOX2-OT \| \| IGHJ3 \| \| AADACL2-AS1 \| \| EIF4EBP3 \| \| IGKV3-7 \| \| ARHGEF26-AS1 \| \| GSTA9P \| \| LINC01998 \| \| PTPRVP \| \| IGKV1-5 \| \| APOBEC3D \| \| WDR86-AS1 \| \| GSTA1 \| \| UGT1A7 \| \| IGKV3-15 \| \| IGKV1-27 \| \| LIFR-AS1 \| \| LINC02453 \| \| BDNF-AS \| \| AF233439.1 \| \| ALDH1L1-AS2 \| \| LINC01096 \| \| AC138904.1 \| \| AC139795.1 \| \| LINC00535 \| \| AC114812.3 \| \| ADH1C \| \| AC004053.1 \| \| LINC02014 \| \| AC120036.1 \| \| AC010280.1 \| \| PCDHAC1 \| \| PRR5-ARHGAP8 \| \| PCP4L1 \| \| NPM1P21 \| \| GLDCP1 \| \| AC018781.1 \| \| LINC00992 \| \| LY75-CD302 \| \| SMIM31 \| \| AC026782.2 \| \| C5orf17 \| \| AC010280.2 \| \| AC022126.1 \| \| AC106799.2 \| \| AC093895.1 \| \| AL691482.3 \| \| LINC02362 \| \| TMEM150C \| \| LINC02198 \| \| AL033397.2 \| \| AC024560.2 \| \| LINC00942 \| \| AC005699.1 \| \| AC083829.1 \| \| AC004704.1 \| \| HS3ST5 \| \| AC119150.1 \| \| GBA3 \| \| CHCHD2P7 \| \| SLC7A11-AS1 \| \| AC069360.1 \| \| AC104806.2 \| \| PCDHA10 \| \| AC010273.3 \| \| RDH10-AS1 \| \| RPSAP70 \| \| AC022101.1 \| \| AC106798.1 \| \| SELENOP \| \| AC106865.1 \| \| AC073475.1 \| \| AC244502.1 \| \| IGKV2D-40 \| \| AC104126.1 \| \| AC093866.1 \| \| AC025539.1 \| \| TUBB7P \| \| LINC00605 \| \| IGKV1D-39 \| \| AC104825.1 \| \| PRB1 \| \| IGLV2-34 \| \| IGHV7-56 \| \| IGHV3-62 \| \| IGLV2-5 \| \| AC100860.1 \| \| IGHV1-67 \| \| IGHV7-34-1 \| \| AC111149.2 \| \| AC008514.1 \| \| LINC02159 \| \| IGHV3-25 \| \| IGLV2-28 \| \| AC044893.1 \| \| IGKV1OR22-1 \| \| IGKV1-13 \| \| MIR3142HG \| \| IGHV3-52 \| \| HOXA-AS2 \| \| ZFHX4-AS1 \| \| GASAL1 \| \| AC027117.1 \| \| LINC01484 \| \| IGHV1-68 \| \| IGHV3-60 \| \| IGLV1-41 \| \| AC007991.2 \| \| PCDHGA11 \| \| IGHV3-19 \| \| PCDHGB4 \| \| CLDN23 \| \| IGHV3-71 \| \| RBPMS-AS1 \| \| CD8B2 \| \| IGHV1-12 \| \| IGHV3-75 \| \| AC022034.4 \| \| IGHV3-42 \| \| PCDHGA3 \| \| ANK3-DT \| \| AC087854.2 \| \| AC120036.3 \| \| AC100854.1 \| \| MPPED2-AS1 \| \| AP003119.1 \| \| IGLL5 \| \| CASP1P2 \| \| SLC25A47P1 \| \| LINC02551 \| \| OR7E158P \| \| AC124276.1 \| \| AF131216.3 \| \| AC103855.3 \| \| SAA2-SAA4 \| \| AC068587.2 \| \| GLYATL1P1 \| \| AP001360.1 \| \| SMIM35 \| \| AC120036.4 \| \| TRIL \| \| IFNG-AS1 \| \| LINC02422 \| \| KLRC4-KLRK1 \| \| AP000812.2 \| \| LINC00346 \| \| CYP2A6 \| \| LINC02446 \| \| USP30-AS1 \| \| AC087235.2 \| \| SALL3 \| \| OR13A1 \| \| CYP2B7P \| \| KLRF2 \| \| SLC5A8 \| \| AC005840.3 \| \| AC084819.1 \| \| PPP1R14B-AS1 \| \| AC084816.1 \| \| HP \| \| AC091078.1 \| \| RPEP6 \| \| CLLU1 \| \| ODC1-DT \| \| AC009318.1 \| \| LINC01619 \| \| LINC02388 \| \| OR7E47P \| \| AC025154.2 \| \| MGAM2 \| \| C1GALT1P1 \| \| AL136418.1 \| \| LINC02300 \| \| AL157871.2 \| \| AL049830.3 \| \| RHOXF1-AS1 \| \| AL157871.3 \| \| NBEAP1 \| \| AL161668.4 \| \| LINC01579 \| \| AL121790.2 \| \| AL049780.2 \| \| LINC02345 \| \| IGHV1OR15-2 \| \| AC025580.1 \| \| LINC01833 \| \| AC036108.1 \| \| IGHV3OR15-7 \| \| AC016705.2 \| \| AC136428.1 \| \| LINC00677 \| \| AC090877.2 \| \| IGHV3OR16-16 \| \| AC007938.1 \| \| AC130456.2 \| \| LINC01992 \| \| IGHV1OR16-3 \| \| AC006960.3 \| \| CSPG4P13 \| \| AC126696.1 \| \| CCDC187 \| \| FRRS1L \| \| AC104024.2 \| \| AQP4-AS1 \| \| LINC02367 \| \| INSYN1-AS1 \| \| FAM157C \| \| MT1L \| \| AC069224.1 \| \| ANKRD20A1 \| \| AL121839.2 \| \| AC009690.1 \| \| AC106799.3 \| \| LINC02178 \| \| AC073476.3 \| \| LMO7-AS1 \| \| AC092142.1 \| \| MUC22 \| \| AC034105.5 \| \| AC005586.2 \| \| AC099518.2 \| \| AL035425.3 \| \| AC012645.3 \| \| AC109446.3 \| \| TUBB8 \| \| LINC02605 \| \| GOLGA6L7 \| \| AC244090.3 \| \| AC093627.7 \| \| AC007342.4 \| \| AC044798.2 \| \| IGHV3OR16-15 \| \| AC092718.6 \| \| MYMX \| \| AC004494.1 \| \| MIR193BHG \| \| CCER2 \| \| LINC01979 \| \| AC007342.5 \| \| AC005695.1 \| \| AC007220.1 \| \| AC004584.3 \| \| MSMB \| \| AC009716.1 \| \| AC015908.3 \| \| SNX19P3 \| \| PPIAP54 \| \| TTC39C-AS1 \| \| AC145207.8 \| \| AC005722.4 \| \| AC011825.4 \| \| AGAP12P \| \| LRRC37A7P \| \| PGDP1 \| \| AC010754.1 \| \| CYP4F35P \| \| ESRG \| \| AP006219.1 \| \| AC009831.2 \| \| LINC00683 \| \| AC005838.2 \| \| AC024267.6 \| \| AC129492.4 \| \| LINC01841 \| \| TCF4-AS1 \| \| AC005336.1 \| \| AP005264.1 \| \| SCAT1 \| \| ZNF790-AS1 \| \| AC011481.2 \| \| AC008649.1 \| \| AC022706.1 \| \| KCNJ2-AS1 \| \| LINC01926 \| \| AC016582.3 \| \| AC016229.2 \| \| AC024592.2 \| \| ZSCAN5DP \| \| CCDC177 \| \| AC139769.2 \| \| AC243960.1 \| \| AC007785.3 \| \| AC123912.4 \| \| AL121761.1 \| \| AC245128.3 \| \| ESPNP \| \| AC007842.1 \| \| AC006262.2 \| \| BICRA-AS1 \| \| AL391001.1 \| \| AC125494.2 \| \| Z93241.1 \| \| AP001172.1 \| \| LINC01480 \| \| IGKV1OR2-11 \| \| AC135506.1 \| \| IGHV3OR16-12 \| \| IGHV3OR16-9 \| \| AC079610.1 \| \| AC091133.6 \| \| LINC00221 \| \| IGHV3OR16-6 \| \| IGHV3OR16-8 \| \| IGHV1OR15-3 \| \| AC087783.1 \| \| AC134879.2 \| \| AL109936.2 \| \| Z97200.1 \| \| AL161716.1 \| \| AL136984.1 \| \| AL365181.2 \| \| AC244517.1 \| \| FGF14-AS2 \| \| AC116351.2 \| \| AL513477.2 \| \| AC254633.1 \| \| AC005162.3 \| \| DOC2B \| \| GAS6-DT \| \| AP005137.2 \| \| AL445423.1 \| \| AC099568.2 \| \| U62317.3 \| \| FAM106A \| \| DGCR9 \| \| GRIN2B \| \| AC092535.5 \| \| AC069148.1 \| \| AC090912.3 \| \| TM4SF19-TCTEX1D2 \| \| AC012313.9 \| \| FAM27E3 \| \| ADRA2B \| \| AL049757.1 \| \| ZNF280B \| \| AL353748.2 \| \| AL161431.1 \| \| IGHV3-54 \| \| AC009318.4 \| \| AC243965.2 \| \| XKR5 \| \| AL390755.1 \| \| AC125603.4 \| \| AC007786.2 \| \| CCL14 \| \| AC005393.1 \| \| IGKV1D-13 \| \| DACH1 \| \| AC005840.4 \| \| IGHV4-4 \| \| AL354718.3 \| \| AC023510.2 \| \| AC002401.4 \| \| HYDIN2 \| \| AC243829.4 \| \| IGHV1OR21-1 \| \| GOLGA6L6 \| \| GPR179 \| \| AC008759.3 \| \| GOLGA6L22 \| \| NLRP3P1 \| \| AC010332.1 \| \| AL161669.3 \| \| IGLV2-8 \| \| PRICKLE4 \| \| IGHV3-41 \| \| AC010998.3 \| \| AL161645.1 \| \| AC100847.1 \| \| AC009878.1 \| \| IGKV1D-12 \| \| BANCR \| \| AC073592.2 \| \| AL590226.2 \| \| AC009159.4 \| \| CR392039.3 \| \| AL513497.1 \| \| AL928654.4 \| \| AL391005.1 \| \| AC007342.8 \| \| AC018892.3 \| \| AC026748.1 \| \| AL031595.1 \| \| AC110771.1 \| \| AL390755.2 \| \| AC007342.9 \| \| AC133065.3 \| \| AC092437.1 \| \| AC018470.1 \| \| AL049536.1 \| \| AL356481.3 \| \| AC004808.2 \| \| TRG-AS1 \| \| AC135068.8 \| \| AC135068.9 \| \| SAMD12-AS1 \| \| LINC01144 \| |

Supplementary Table 2. TCGA Identifiers

| TCGA ID (n=121) |
| --- |
| TCGA-CV-7440-01A-11R-2132-07 |
| TCGA-BA-4076-01A-01R-1436-07 |
| TCGA-CR-7371-01A-11R-2016-07 |
| TCGA-CV-7090-01A-11R-2016-07 |
| TCGA-CR-7391-01A-11R-2016-07 |
| TCGA-CV-7180-01A-11R-2016-07 |
| TCGA-BA-6873-01A-11R-1873-07 |
| TCGA-CV-6945-01A-11R-1915-07 |
| TCGA-BA-5557-01A-01R-1514-07 |
| TCGA-CN-4737-01A-01R-1436-07 |
| TCGA-CQ-7065-01A-11R-2081-07 |
| TCGA-CR-7372-01A-11R-2016-07 |
| TCGA-CV-5979-01A-11R-1686-07 |
| TCGA-CV-7255-01A-11R-2016-07 |
| TCGA-CV-7416-01A-11R-2081-07 |
| TCGA-DQ-5624-01A-01R-1873-07 |
| TCGA-CN-5365-01A-01R-1436-07 |
| TCGA-CV-7177-01A-11R-2016-07 |
| TCGA-CV-7177-11A-01R-2016-07 |
| TCGA-CV-7424-11A-01R-2081-07 |
| TCGA-CV-7424-01A-11R-2081-07 |
| TCGA-CN-6997-01A-11R-2016-07 |
| TCGA-CN-4739-01A-02R-1514-07 |
| TCGA-CR-7370-01A-11R-2132-07 |
| TCGA-CN-4723-01A-01R-1436-07 |
| TCGA-CV-5432-01A-02R-1686-07 |
| TCGA-CV-6962-11A-01R-1915-07 |
| TCGA-CV-6962-01A-11R-1915-07 |
| TCGA-CN-6023-01A-11R-1686-07 |
| TCGA-CV-7421-01A-11R-2081-07 |
| TCGA-CR-7374-01A-11R-2016-07 |
| TCGA-CN-5360-01A-01R-1436-07 |
| TCGA-BB-4217-01A-11R-2081-07 |
| TCGA-CN-6012-01A-11R-1686-07 |
| TCGA-CR-7402-01A-11R-2016-07 |
| TCGA-CR-7388-01A-11R-2016-07 |
| TCGA-CR-7364-01A-11R-2016-07 |
| TCGA-CV-6935-01A-11R-1915-07 |
| TCGA-CV-6935-11A-01R-1915-07 |
| TCGA-CV-7101-01A-11R-2016-07 |
| TCGA-CV-7101-11A-01R-2016-07 |
| TCGA-CV-7437-11A-01R-2132-07 |
| TCGA-CQ-5326-01A-01R-1873-07 |
| TCGA-IQ-7632-01A-11R-2081-07 |
| TCGA-CQ-6223-01A-11R-1915-07 |
| TCGA-DQ-7588-01A-11R-2081-07 |
| TCGA-CQ-5334-01A-01R-1686-07 |
| TCGA-CN-4726-01A-01R-1436-07 |
| TCGA-CV-6940-01A-11R-1915-07 |
| TCGA-CQ-6220-01A-11R-1915-07 |
| TCGA-CV-6948-01A-11R-1915-07 |
| TCGA-CQ-5332-01A-01R-1686-07 |
| TCGA-CV-7102-01A-11R-2016-07 |
| TCGA-CV-7235-01A-11R-2016-07 |
| TCGA-CV-7235-11A-01R-2016-07 |
| TCGA-CV-6936-11A-01R-1915-07 |
| TCGA-CV-6936-01A-11R-1915-07 |
| TCGA-CQ-6228-01A-11R-1915-07 |
| TCGA-CN-6995-01A-31R-2016-07 |
| TCGA-CV-5436-01A-01R-1514-07 |
| TCGA-CQ-7068-01A-11R-2081-07 |
| TCGA-CV-6953-01A-11R-1915-07 |
| TCGA-CV-7407-01A-11R-2081-07 |
| TCGA-CN-5369-01A-01R-1436-07 |
| TCGA-BA-5558-01A-01R-1514-07 |
| TCGA-CV-7413-01A-11R-2081-07 |
| TCGA-CN-4740-01A-01R-1436-07 |
| TCGA-CN-4729-01A-01R-1436-07 |
| TCGA-CX-7082-01A-11R-2016-07 |
| TCGA-H7-7774-01A-21R-2081-07 |
| TCGA-CV-7432-01A-11R-2132-07 |
| TCGA-CV-7432-11A-01R-2132-07 |
| TCGA-CV-6937-01A-11R-2016-07 |
| TCGA-CR-7379-01A-11R-2016-07 |
| TCGA-CN-6994-01A-11R-1915-07 |
| TCGA-CV-7427-01A-11R-2081-07 |
| TCGA-CV-7095-01A-21R-2016-07 |
| TCGA-CR-7386-01A-11R-2016-07 |
| TCGA-CR-7395-01A-11R-2016-07 |
| TCGA-CR-7373-01A-11R-2016-07 |
| TCGA-CV-7099-01A-41R-2016-07 |
| TCGA-CV-7414-01A-11R-2081-07 |
| TCGA-CV-7423-11A-01R-2081-07 |
| TCGA-CV-7423-01A-11R-2081-07 |
| TCGA-CR-6484-01A-11R-1873-07 |
| TCGA-CV-6942-01A-21R-2016-07 |
| TCGA-CV-6955-11A-01R-2016-07 |
| TCGA-CV-6955-01A-11R-2016-07 |
| TCGA-CV-7236-01A-11R-2016-07 |
| TCGA-CV-6952-01A-11R-1915-07 |
| TCGA-CN-4736-01A-01R-1436-07 |
| TCGA-D6-6825-01A-21R-1915-07 |
| TCGA-CN-5370-01A-01R-2016-07 |
| TCGA-CV-5977-01A-11R-1686-07 |
| TCGA-CX-7085-01A-21R-2016-07 |
| TCGA-DQ-5630-01A-01R-1873-07 |
| TCGA-CR-6493-01A-11R-1873-07 |
| TCGA-CQ-5330-01A-01R-1686-07 |
| TCGA-CQ-6221-01A-11R-2081-07 |
| TCGA-CQ-6225-01A-11R-1915-07 |
| TCGA-CV-6934-11A-01R-1915-07 |
| TCGA-CV-6934-01A-11R-1915-07 |
| TCGA-BA-4074-01A-01R-1436-07 |
| TCGA-CR-7394-01A-11R-2016-07 |
| TCGA-CR-7390-01A-11R-2016-07 |
| TCGA-CV-6956-11A-01R-2016-07 |
| TCGA-CV-6956-01A-21R-2016-07 |
| TCGA-CR-6488-01A-12R-2081-07 |
| TCGA-CV-7438-11A-01R-2132-07 |
| TCGA-CV-7438-01A-21R-2132-07 |
| TCGA-D6-6515-01A-21R-1873-07 |
| TCGA-HD-7831-01A-11R-2132-07 |
| TCGA-BA-6871-01A-11R-1873-07 |
| TCGA-CR-6478-01A-11R-1873-07 |
| TCGA-CR-7383-01A-11R-2132-07 |
| TCGA-D6-6516-01A-11R-1873-07 |
| TCGA-CN-6018-01A-11R-1686-07 |
| TCGA-CQ-5331-01A-02R-1873-07 |
| TCGA-CV-7437-01A-21R-2132-07 |
| TCGA-BA-4078-01A-01R-1436-07 |
| TCGA-CR-7390-01A-11R-2016-07 |
